# Oncogenic Brain Metazoan Parasite Infection

**DOI:** 10.1155/2013/263718

**Published:** 2013-09-18

**Authors:** Angela N. Spurgeon, Marshall C. Cress, Oroszi Gabor, Qing-Qing Ding, Tomoko Tanaka, Douglas C. Miller

**Affiliations:** ^1^Department of Surgery, Division of Neurological Surgery, School of Medicine, University of Missouri, Columbia, MO, USA; ^2^Department of Pathology, School of Medicine, University of Missouri, Columbia, MO, USA

## Abstract

Multiple observations suggest that certain parasitic infections can be oncogenic. Among these, neurocysticercosis is associated with increased risk for gliomas and hematologic malignancies. We report the case of a 71-year-old woman with colocalization of a metazoan parasite, possibly cysticercosis, and a WHO grade IV neuroepithelial tumor with exclusively neuronal differentiation by immunohistochemical stains (immunopositive for synaptophysin, neurofilament protein, and Neu-N and not for GFAP, vimentin, or S100). The colocalization and temporal relationship of these two entities suggest a causal relationship.

## 1. Introduction

The notion that parasitic infections may be associated with neoplastic growth has existed in the literature for nearly a century [1]. Subsequent studies have suggested and in some situations linked various parasitic infections to multiple neoplasm types including meningiomas [2], primary cerebral rhabdomyosarcoma [3], Burkitt's lymphoma [4], cholangiocarcinoma [5], colon carcinoma [6], hepatocellular carcinoma, and bladder carcinoma [5].

Neurocysticercosis is the most common parasitic disease of the central nervous system. A case control study [7], a large autopsy series [8], and case reports [9–12] have implicated it in the pathogenesis of gliomas. The brain tumors in these reports have been characterized as glioblastoma multiforme, anaplastic astrocytoma, anaplastic oligoastrocytoma, oligodendroglioma, and even pituitary adenoma. Our aim is to report a patient with colocalization of a metazoan parasite, possibly cysticercosis, and a high-grade “glioma” with exclusive neuronal differentiation by immunohistochemical stains.

## 2. Case Report

### 2.1. Initial Admission

**Figure 1:**
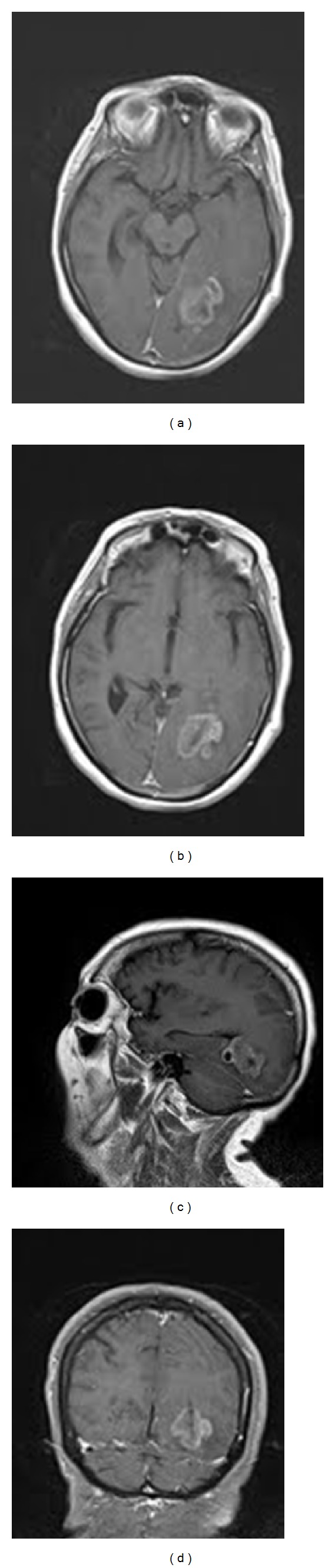
Initial brain MRI. (a), (b) Axial T1-weighted image with gadolinium, showing the enhancing intra- and periventricular left occipitotemporal mass. There is minimal mass effect. In (b) there is a suggestion of a “daughter cyst” lateral to the main lesion. (c) Sagittal T1-weighted image, with gadolinium, showing the lesion mostly within the occipital horn of the lateral ventricle. (d) Coronal T1-weighted image with gadolinium. The abnormal tissue involves the parenchyma around the ventricle as well as within the lumen.

**Figure 2:**
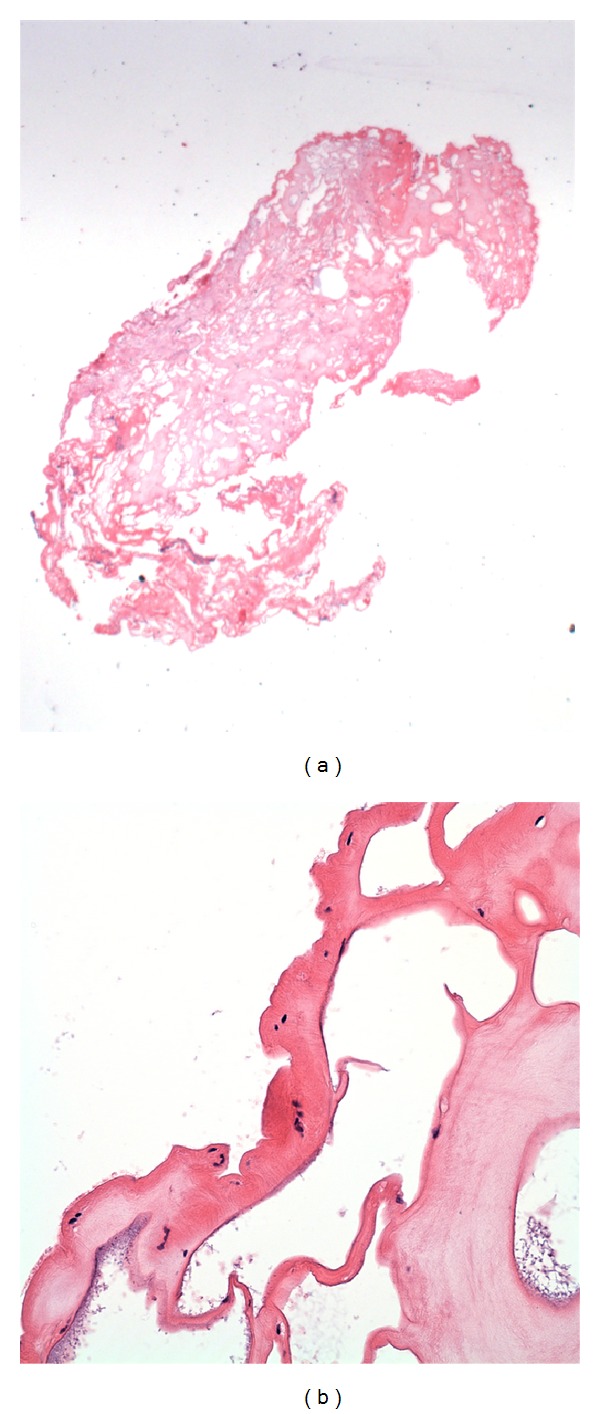
H&E stained sections demonstrating the degenerate parasite after initial craniotomy. (a) The entire tissue is a complex of cysts, with no viable basophilic nuclei apparent at low power, including no inflammatory cells (original magnification 12.5x). (b) At higher magnification, the edge of the abnormal tissue has the typical appearance of a chitinous exoskeleton with a few surviving small nuclei beneath it (original magnification 400x).

This 71-year-old woman presented to the emergency room with confusion and a two-month history of headache and seizure-like episodes. A brain MRI demonstrated an intra- and periventricular occipitotemporal mass (Figure 1). She underwent craniotomy, and a discrete intraventricular mass was removed. The fresh tissue was nearly transparent, firm, and rubbery. Histopathological examination revealed a degenerate metazoan larval parasite; the exact identification was not possible due to the advanced degeneration of the organism (Figure 2). The resection was halted when the frozen section identified the parasite, so a gross total resection was not achieved. Postoperative investigations included an ELISA for serum antibodies to *Taenia solium*, which was negative. Given the presence of only one cyst, further antiparasitic therapy was not administered. Her postoperative course was uneventful, and she was discharged to a rehabilitation facility on hospital day eight. 

### 2.2. Second Admission

**Figure 3:**
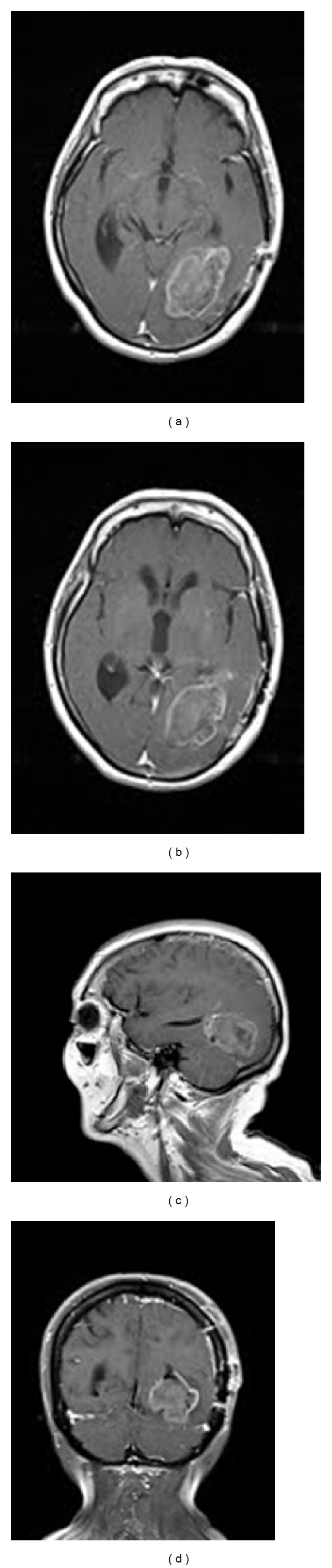
Brain MRI at second admission. (a), (b) Axial T1-weighted image with gadolinium, showing a larger mass with peripheral enhancement compared to the original appearance of the lesion as seen in Figure 1. (c) Sagittal T1-weighted image with gadolinium, showing the larger lesion with a more substantial intraparenchymal component. (d) Coronal T1-weighted image with gadolinium also shows the enlarged lesion with greater mass effect.

**Figure 4:**
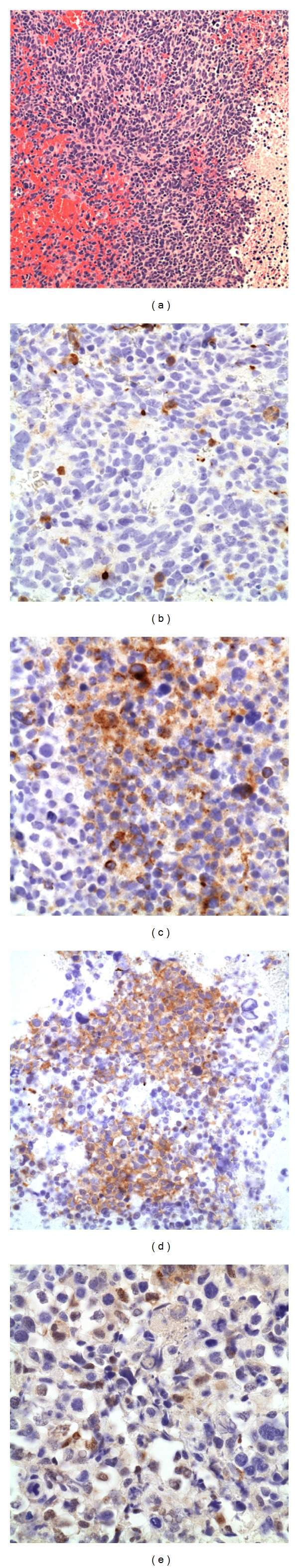
Tissue removed from the second craniotomy. (a) The tumor is a densely cellular lesion composed mostly of small cells with small cell bodies (H&E, original magnification 200x). (b) A GFAP immunostain marks scattered single cells representing entrapped reactive astrocytes, 600x. (c) A Synaptophysin immunostain labels the cytoplasm of multiple tumor cells, surrounding their centrally-located nuclei (600x). (d) A neurofilament protein immunostain also labels the cytoplasm of many tumor cells as well as some processes from them (400x). (e) A Neu-N immunostain distinctly labels the nuclei of many tumor cells (600x).

Five weeks after discharge, she began to display substantial cognitive difficulty. At readmission, a brain MRI demonstrated enlargement of the left temporal occipital abnormality (Figure 3). Repeat craniotomy was undertaken, revealing a high-grade glial tumor. Permanent sections from the second craniotomy showed a WHO grade IV neuroepithelial tumor which was composed of small to medium-size cells with exclusively neuronal differentiation by immunohistochemical stains (Table 1 and Figure 4). The tumor had multiple foci with necrosis, justifying a WHO grade of IV (not shown). A residual intraventricular component, submitted with the tumor tissue, was seen again to be a portion of a degenerate larval parasite.

A ventriculoperitoneal shunt was placed on hospital day eight. She was discharged on hospital day thirteen with significant speech impairment to a skilled nursing facility. She succumbed to complications related to her disease nine months after diagnosis; however, a brain MRI did not show recurrent tumor at the time of her final presentation.

## 3. Discussion

We report the rare occurrence of a case with coexistent parasitic disease and a high-grade glioma. The exact nature of the parasite, as noted, could not be determined histologically due to the advanced degeneration of its tissues. Cysticercosis antibody testing was negative, but serological tests for cysticercosis by ELISA have a sensitivity of only 70% and a specificity of only 50%. The patient did not have any risk factors for neurocysticercosis, so infection with a different Metazoan parasite endemic to Missouri was certainly possible, such as *Baylisascaris procyonis*, which is known to infect raccoons. *Baylisascaris* infections are not yet described in association with tumors but this may reflect their relative rarity. Further testing was not performed in this case because it was felt that the results would not change treatment decisions. 

While association between parasitic infections and gliomas are documented in the literature, it is not entirely clear whether parasitic infections predispose to the development of gliomas or whether the inverse is true and gliomas predispose a host to parasitic infections; however, the available literature does tend to favor the theory that the parasitic infection has an oncogenic effect. 

The close colocalization of the tumor and the parasite in this case supports a causal relationship between the development of the neoplasm and the parasitic infection. In a case-control study by Del Brutto et al. [7], of eight patients with glioma and a history of neurocysticercosis, six had colocalized disease. Of three other case reports published in the English language of concomitant CNS parasitic infections and glioma, colocalization was difficult to determine in one [13] but in the other two [10, 12] there was colocalization. Of course, it is also possible that the association of the parasitic infestation and the glioma is a coincidental association, but there are reasons to find a causal relationship plausible. One reason, as just noted, is the spatial proximity of the tumor to the larval cyst.

The likely temporal relationship between the tumor and parasitic cyst is a second reason to suggest a causal relationship. The advanced degeneration of the parasite's tissues make it likely that it was present for years and that the tumor arose at a later date. In the case-control study by Del Brutto et al. [7], two of eight patients had a prior history of CNS parasitic disease before their diagnoses with gliomas. 

Plausible mechanisms to explain this oncogenic effect include parasite-induced immune dysregulation, transfer of genetic material between parasite and host, and chronic inflammation. Furthermore, potential mechanisms may independently exert a carcinogenic effect or a combination of them may be at play [14]. To lend support to the immune dysregulation theory, Herrera et al. [13] demonstrated increased DNA damage in peripheral lymphocytes from patients infected with cysticerci, suggesting a causal mechanism for the observed increased risk in infested patients for hematologic malignancies. Later they showed DNA damage in cultured lymphocytes treated with a soluble factor extracted from *Taenia solium* [15]. With regard to chronic inflammation, gliomas have been known to develop at sites of chronic inflammation. Sabel et al. [16] reported a case of a glioblastoma occurring at the site of a previous penetrating metal splinter injury sustained 19 years before presentation. The resected tumor contained chronic abscess sites. The authors hypothesized that metallic splinters and chronic infection might have produced a proliferative stimulus inciting neoplastic transformation of reactive glial cells secondary to the persistently active chronic inflammation. 

The exclusive neuronal differentiation of the tumor, based on the results of immunohistochemical stains, is also a unique feature in this case. Only one of the prior case reports associating gliomas with cysticercosis in the English literature has been reported on any immunostain evaluation of the tumor; in that case it was only a positive glial fibrillary acidic protein immunostain in an anaplastic astrocytoma [10]. Neuronal components in glial tumors are increasingly being recognized with wider use of larger immunohistochemical patterns (as well as molecular genetic analyses) but the prognostic implications of a purely neuronal tumor, such as the one we have encountered, remain unknown. 

## 4. Conclusions

We present a case of a patient with a metazoan parasitic infection, possibly cysticercosis and a high-grade glioma with exclusive neuronal differentiation on immunohistochemical stains. The colocalization and temporal relationship of these two entities suggest a causal relationship. The mechanism by which a parasitic infection produces an oncogenic effect is likely multifactorial and should be a topic for future research. The implications of a WHO grade IV neoplasm with purely neuronal differentiation are not clear. 

## Figures and Tables

**Table 1:** Summary of immunohistochemical stains from the second craniotomy.

Test	Result
Synaptophysin	Positive
Neurofilament protein*	Positive
Neu-N	Positive
Vimentin	Negative
GFAP	Negative
S100	Negative

*Intermediate molecular weight NFP, antibody RMDO20.
